# Effect of Multiplication and Charge Layers on the Gain in InGaAsSb/AlGaAs Avalanche Photodiodes at Room Temperature

**DOI:** 10.3390/s25072255

**Published:** 2025-04-03

**Authors:** Tetiana Manyk, Jarosław Rutkowski, Krzysztof Kłos, Nathan Gajowski, Sanjay Krishna, Piotr Martyniuk

**Affiliations:** 1 Institute of Applied Physics, Military University of Technology, 2 Kaliskiego St., 00-908 Warsaw, Poland; tetjana.manyk@wat.edu.pl (T.M.); jaroslaw.rutkowski@wat.edu.pl (J.R.); 2 Photin sp. Z O.O, 15 Lutosławskiego St., 05-080 Klaudyn, Poland; 3 Department of Electric Engineering, The Ohio State University, 2024 Neil Avenue, Columbus, OH 43210, USA; gajowski.2@osu.edu (N.G.); krishna.53@osu.edu (S.K.)

**Keywords:** SWIR, IR detectors, avalanche detectors, APDs, impact multiplication gain, InGaAsSb, AlGaSb

## Abstract

This paper presents a theoretical analysis of npBp infrared (IR) barrier avalanche photodiode (APD) performance operating at 300 K based on a quaternary compound made of A^III^B^V^—InGaAsSb, lattice-matched to the GaSb substrate with a *p*-type barrier made of a ternary compound AlGaSb. Impact ionization in the multiplication layer of InGaAsSb separate absorption, grading, charge, and multiplication avalanche photodiodes (SAGCM APDs) was studied using the Crosslight Software simulation package APSYS. The band structure of the avalanche detector and the electric field distribution for the multiplication and absorption layers were determined. The influence of the multiplication and charge layer parameters on the impact multiplication gain and the excess noise factor was analyzed. It has been shown that with the decrease in the charge layer doping level, the gain and the breakdown voltage increase, but the punch-through voltage decreases, and the linear range of the APD operating voltages widens. The multiplication layer doping level slightly affects the detector parameters, while increasing its width, the photocurrent and the breakdown voltage also increase. The detector structure proposed in this work allows us to obtain a comparable gain and lower dark currents to the APD detectors made of InGaAsSb previously presented in the literature. The performed simulations confirmed the possibility of obtaining APDs with high performance at room temperatures made of InGaAsSb for the SWIR range.

## 1. Introduction

Avalanche photodiodes (APDs) based on InGaAs/InAlAs materials have attracted much attention in recent years and are widely used in many fields, including the most important one—optical communication systems—due to their response time and high sensitivity [1,2,3,4,5]. In the past decades, the separated absorption, grading, charge, and multiplication (SAGCM) structure A^III^B^V^ APDs have been intensively researched, since the electric field in the absorption and multiplication layers can be precisely adjusted. In this type of structure, at a voltage called punch-through (*U_pt_*), the depletion region begins to expand into the absorber and, thanks to the electric field, minority carriers are transported toward the junction, causing a significant increase in the photocurrent. Carrier avalanche multiplication begins at this voltage and continues up to the breakdown voltage (*U_br_*), at which a further avalanche current increase occurs [4].

Like other semiconductor devices, the first commercialized APDs were fabricated based on Si due to the long-term investment into Si processing technology. After the commercialization of Si APDs, a second major type of marketed APD was made of InGaAs, with InP or InAlAs multipliers and wavebands ranging from 1 to 1.7 μm [5,6,7,8,9,10,11,12,13]. Recently, more attention has been paid to APDs based on A^III^B^V^ quaternary compounds such as InGaAsSb, AlGaAsSb, and AlInAsSb, which may be used in future optical communications, type-II superlattice (T2SLs, “Ga-based” and “Ga-free”), and 2D material-based IR APDs.

The quaternary systems InGaAsSb have great importance for various civil and military applications, including atmospheric remote sensing, and industrial areas, such as gas detection [14,15,16,17,18]. These materials have found wide application due to their cut-off wavelength flexibility by changing the molar composition of In and As. The advantages of the tested InGaAsSb detectors on a GaSb substrate are a lower dark current and higher quantum efficiency than the ternary InGaAs detectors at a temperature of 300 K and a cut-off wavelength (50% cut-off) of 2.5 μm [19]. Recently, the combination of a ternary InGaAs absorber and a quaternary AlGaAsSb multiplication region has been described, which provides low excess noise and a low dark current by the lattice matching of the tested materials, which next results in a high-current-gain operation [20,21,22].

The theoretical analysis of *I*-*V* characteristics for npBp avalanche barrier detectors with an active layer based on quaternary bulk materials (In_0.14_Ga_0.86_As_0.10_Sb_0.90_) and a ternary barrier layer (Al_0.20_Ga_0.80_Sb) at room temperature is presented in our work. So far, the published articles have presented research on these detectors based on the molar composition of indium (In) of *x*_In_ = 0.28 and on the molar composition of arsenic (As) *x*_As_ = 0.10. Reducing the In fraction to *x*_In_ = 0.14 in the quaternary tested materials shifts the sensitivity edge to 2.2 µm and reduces the dark current. In this article, the charge and multiplication layer doping/thickness influence on the *I*-*V* characteristics is presented. A quaternary material with this *x*_In_ = 0.14 and *x*_As_ = 0.10 composition is promising for SWIR range applications.

## 2. APD Structure and Simulation Models

Crosslight simulators are based on finite element analysis in two or three dimensions. They involve a large number of sophisticated physical and numerical models. APSYS is a general purpose 2D/3D modeling software program for semiconductor devices. APSYS is based on finite element analysis, and it includes many advanced physical models such as hot carrier transport, heterojunction models, and thermal analysis. APSYS offers a very wide range of applications and can handle almost all semiconductor devices. APSYS offers a simulation environment for modern semiconductor devices [23]. The material data used in the modeling are given in [24,25,26,27,28,29,30] and are presented in detail in Ref. [31]. In this study, we simulate the operation of the APD detectors in two dimensions (2D).

The avalanche multiplication gain, *M*, was determined as the ratio of the photocurrent at a given voltage to the photocurrent corresponding to the punch-through voltage *U*_pt_ (the unity-gain condition is normally identifiable as a plateau in the photocurrent for ≥*U*_pt_). The excess noise factor was determined based on the following relation:

(1) $$F_{e}\left(M\right)=M\left[1-\left(1-k\right){\left[\frac{M-1}{M}\right]}^{2}\right],$$

where *k* is the ratio of the avalanche ionization coefficient rate of holes to electrons. The Chynoweth model [32] was used to simulate the electron and hole ionization coefficients:

(2) $$\alpha_{e}=a_{e}e^{\frac{b_{e}}{E}},$$

(3) $$\alpha_{h}=a_{h}e^{\frac{b_{h}}{E}},$$

where *E* is the electric field in the multiplication layer. The *k* coefficient varied, with the change in gain *M* being dependent on *E*. The InGaAsSb material parameters used in the simulations are presented in Table 1.

**Figure 1:**
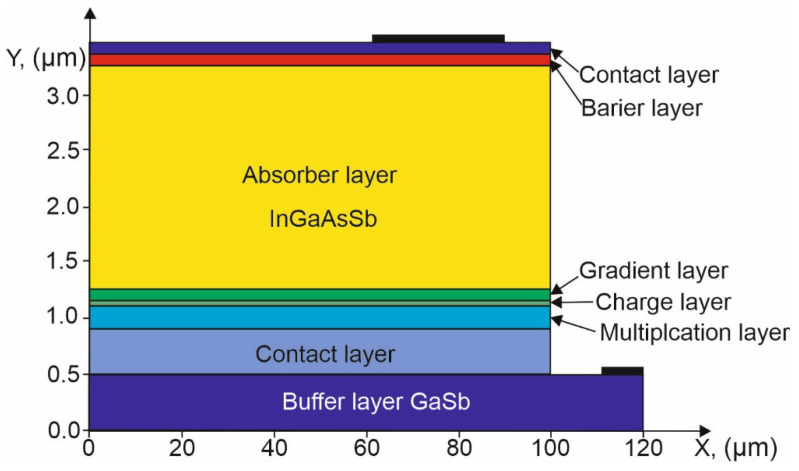
Structure of the analyzed npBp barrier APD.

This paper analyzes the performance of npBp barrier APD IR detectors based on a quaternary compound InGaAsSb made of A^III^B^V^ materials, lattice-matched to a binary GaSb substrate. The structure included the buffer GaSb layer, a lower contact and multiplication In_0.05_Ga_0.95_As_0.10_Sb_0.90_ layer (ML), a charge layer (CL), a gradient layer (GL), an absorber layer (AL) made of the In_0.14_Ga_0.86_As_0.10_Sb_0.90_, and a barrier layer (BL) made of a ternary Al_0.20_Ga_0.80_Sb material with and the upper contact layer built of GaSb. The barrier layer was used to limit the injection of minority carriers from the upper contact side. Its presence reduces the dark current but does not affect the APD operation as much as the multiplication and charge layers. The cross-sections of the 2D structure of the analyzed detector are shown in Figure 1, and the main parameters, described in detail, are presented in Table 2.

**Figure 2:**
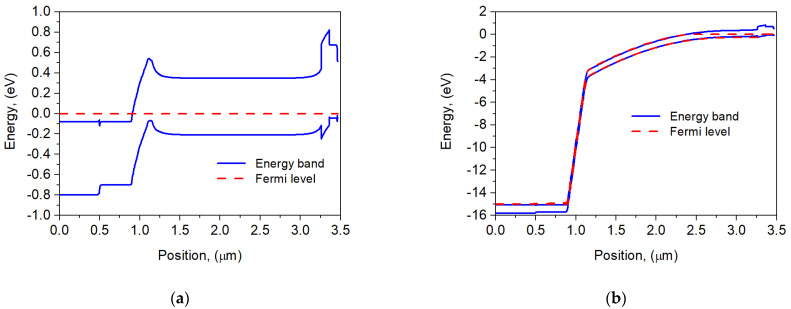
Band structure of the APD barrier detector at 300 K for *U* = 0 V (**a**) and *U* = 15 V (**b**). The position of the Fermi level has been marked with a dashed line.

The theoretical simulation energy band profile of the APD is shown in Figure 2. In this structure, an electron barrier is created, allowing for the transport of minority carriers (holes) from the AL to the upper CL while blocking electron transport. However, in the analyzed case, there is also a small potential barrier in the conduction band near the p-n junction, which may block the flow of minority electrons from the AL to the p-n junction and thus reduce the photocurrent. This barrier vanishes at reverse bias *U* = 15 V [punch-through voltage at which the depletion region extends through the charge layer to the edge of the AL (see dashed Fermi line in Figure 2b)]. Below the punch-through voltage, the photocurrent is typically low because many of the photogenerated carriers in the AL do not have sufficient energy to cross the heterojunction interfaces.

## 3. Results and Discussion

**Figure 3:**
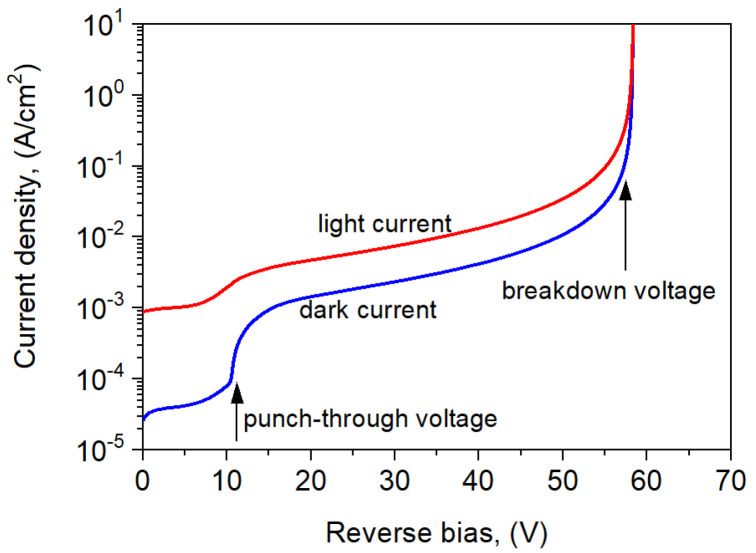
Theoretical simulation of the dark current and photocurrent densities for the reverse-biased APD at *T* = 300 K. Incident radiation: power *p* = 10 W and wavelength *λ* = 1.55 μm.

The influence of the detector layer parameters on the dark current and the photocurrent was analyzed. Numerical simulations were performed for incident radiation with power *p* = 0.1 − 10 W and wavelength *λ* = 1.55 μm. Figure 3 shows the dark and light currents under the reverse bias at *T* = 300 K. The punch-through voltage (reverse) was estimated at the level of 10.5 V, and the breakdown voltage was 58.4 V. When the APD reaches the punch-through voltage, the p–n-junction depletion begins to extend to the InGaAsSb AL, the charge layer barrier in the conduction band vanishes, and the photogenerated carriers move to the ML. The photocurrent for *U* ≤ *U_pt_* results from the optical generation of carriers in the contact and the multiplication layers.

**Figure 4:**
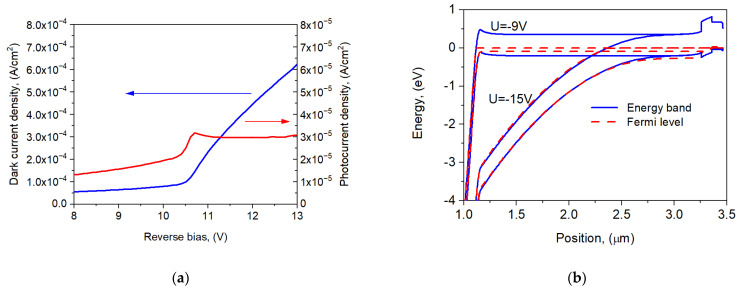
Dark current and photocurrent versus voltage near *U_pt_* (**a**) and the AL band structure for *U* = −9 V and *U* = −15 V (**b**) at *T* = 300 K. The position of the Fermi level has been marked with a dashed line.

The precise determination of the punch-through voltage requires analysis of the detector band structure and *I*-*V* characteristics. Figure 4 shows the dependence of the dark current and photocurrent on the voltage near *U_pt_* (Figure 4a) and the shape of the energy bands and the position of the quasi-Fermi levels at the reverse bias of 9 V and 15 V. The punch-through effect on the *I*-*V* characteristics appears at a reverse bias of 10.5 V. Below this voltage (for *U* = −9 V), a small barrier in the conduction band was observed, which disappeared at voltages above −11 V, causing a significant increase in the photocurrent. At the same time, the splitting of the quasi-Fermi levels increases, which at a voltage of −15 V reaches a value comparable to the absorber energy gap. This indicates the expansion of the depletion region to the entire AL. Also, part of the electric field drops in the absorber region, facilitating the transport of electrons to the multiplication region. When the operating voltage is between −15 V and −58.4 V, the APD operates in a linear mode. The number of carriers being multiplicated increases and the photocurrent is multiplied, with a gain of up to 200. When the APD operates between *U*_pt_ and 95% of *U*_br_, the photocurrent increases from 3.3 × 10^−3^ A/cm^2^ to 9.7 × 10^−2^ A/cm^2^, while the dark current increases from 7.8 × 10^−4^ A/cm^2^ to 2.7 × 10^−2^ A/cm^2^.

**Figure 5:**
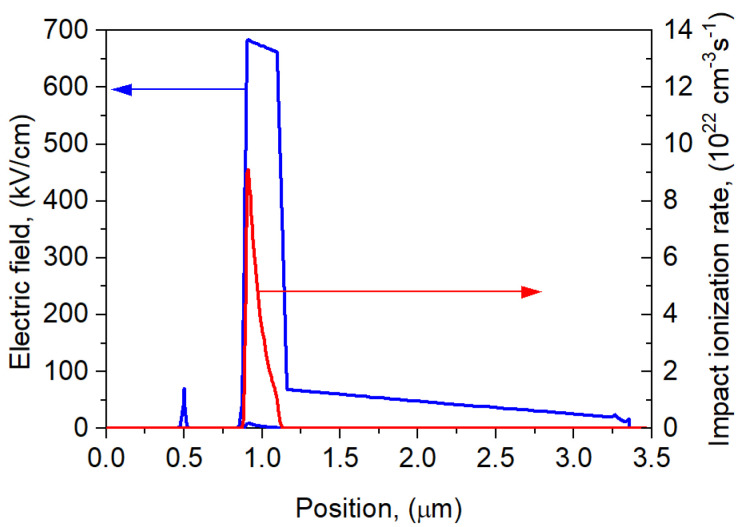
Electric field and impact ionization distribution along npBp detector at *U* = −55.5 V at *T* = 300 K.

An analysis of the electric field distribution and impact ionization rate was performed. Figure 5 shows the comparison of the electric field distribution and impact ionization rate for the npBp APD with the parameters presented in Table 2 at a reverse bias of 0.95 × *U*_br_ = −55.5 V.

The electric field distribution is typical for APDs. A high field of the order of 700 kV/cm occurs only in the ML, while in the charge, gradient, and absorber layers, there is a 10-times-lower field causing a drift of minority carriers (electrons) towards the ML and the p-n junction. Impact ionization occurs mainly in the ML region near the p-n junction.

**Figure 6:**
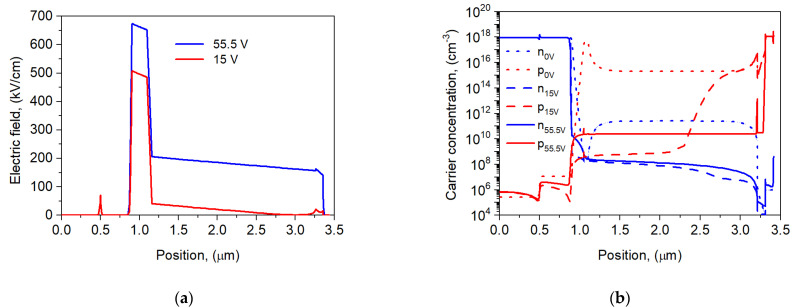
Electric field distribution (**a**) and electron and hole concentrations at *U* = −15 V and *U* = −55.5 V (**b**) for the npBp APD at 300 K. The position of the equilibrium concentration has been marked with a dotted line.

To assess the impact of the ionization contribution, the electric field and carrier concentration distribution at the *U*_pt_ and 0.95 × *U*_br_ reverse bias conditions were analyzed and are presented in Figure 6. The electric field (see Figure 6a) in the AL increases faster than that in the ML. The electron and hole concentrations increased versus the reverse bias (Figure 6b). On the other hand, in the depleted absorber, the photogenerated electrons and the multiplied holes from the ML significantly affect the transport characteristics.

**Figure 7:**
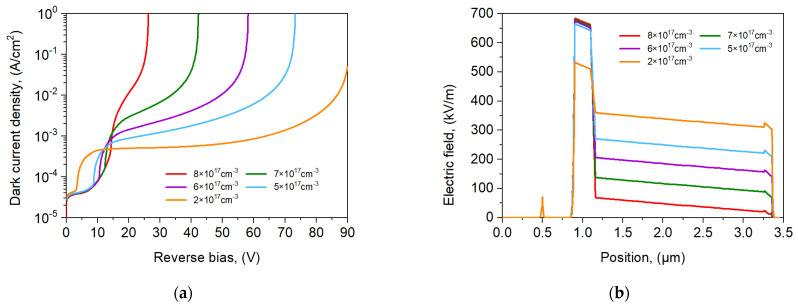
*I*-*V* characteristics (**a**) and electric field distribution at 0.95 × U_br_ (**b**) for npBp APD at *T* = 300 K with selected charge layer doping *p* = 2 × 10^17^, 5 × 10^17^, 6 ×10^17^, 7 × 10^17^, 8 × 10^17^ cm^−3^.

The influence of the charge layer doping on the dark current and electric field distribution for the npBp APD was analyzed, and the results are presented in Figure 7. Figure 7a shows that with higher CL doping, the *U*_br_ is lower but the *U*_pt_ is higher and the linear part of the *I*-*V* characteristic is reduced. This is connected with the decrease in the electric field in the ML versus CL doping, as shown in Figure 7b. The charge layer serves as an electric field control layer, ensuring that the electric field in the ML is sufficient to reach a high gain while simultaneously reducing the field in the AL below the tunneling threshold to maintain a low dark current.

**Figure 8:**
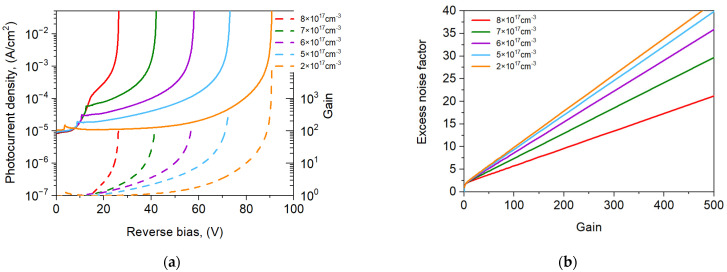
Photocurrent gain (**a**) and excess noise factor (**b**) for npBp APD at 300 K with selected charge layer doping *p* = 2 × 10^17^, 5 × 10^17^, 6×10^17^, 7 × 10^17^, 8 × 10^17^ cm^−3^.

The CL doping significantly affects the photocurrent and APD’s gain. Figure 8a shows the theoretical simulation of the photocurrent density versus the reverse bias for CL selected doping at 300 K. The increase in the CL doping causes an increase in the photocurrent but decreases *U*_br_ (Figure 8a). For voltages near *U*_br_ (0.95 × *U*_br_), the gain varied from 30 to 40, with the charge layer doping varying from 8 × 10^17^ cm^−3^ to 2 × 10^17^ cm^−3^. The dependence of the electric field *E* on the applied voltage was determined, and then the dependence of the *k* coefficient on the bias was extracted. We can see that in the avalanche multiplication range, the *k* value changed from 0.02 to 0.08. The *k* and *M* allowed us to determine the excess noise factor F(M) according to the relation (1). F(M) is presented in Figure 8b for selected levels of charge layer doping. The excess noise factor increases versus gain and lowering the doping level of the charge layer.

**Figure 9:**
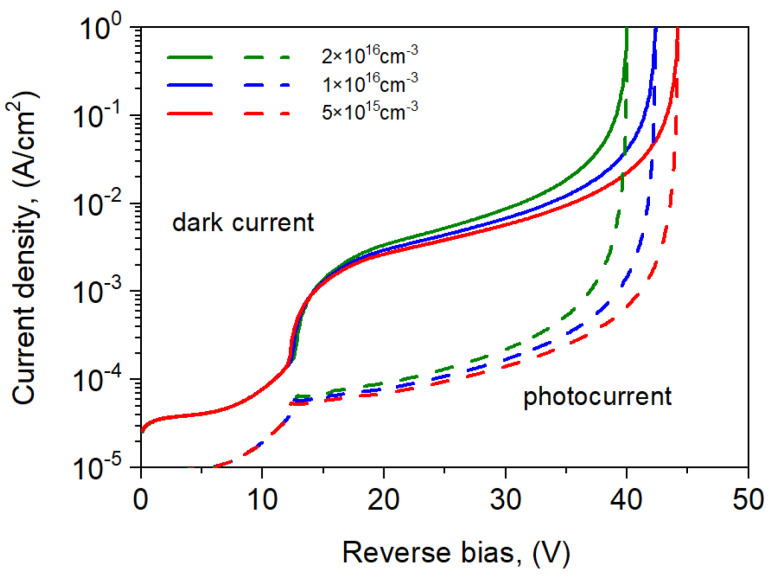
Dark current and photocurrent densities for the npBp APD at *T* = 300 K for selected ML doping: *p* = 5 × 10^15^, 1 × 10^16^, 2 × 10^16^ cm^−3^.

The influence of the ML parameters on the *I*-*V* characteristics and photocurrent gain was also analyzed. Figure 9 shows the influence of the ML doping level on the dark current and photocurrent for the tested npBn detector at 300 K. An increase in the dopant concentration in the ML by one order causes a slight increase in gain and a decrease in the avalanche breakdown voltage *U*_br_ by several volts. This effect is caused by the electric field increase in the absorber region at the expense of the reduction in the field in the ML.

**Figure 10:**
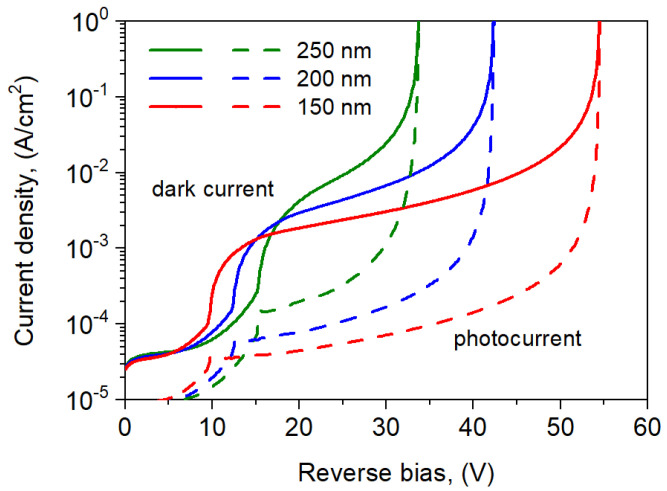
Dark current and photocurrent densities for the npBp APD at *T* = 300 K for selected ML thickness: *d* = 150, 200, and 250 nm.

The influence of the ML thickness on the avalanche multiplication rate is more pronounced. The simulated *I*-*V* characteristics for the selected ML thicknesses are shown in Figure 10. The punch-through voltage *U*_pt_ increases versus ML width, while the breakdown voltage decreases. The photocurrent and the avalanche gain increase versus ML thickness, but at the same time the voltage range of the ML decreases.

**Figure 11:**
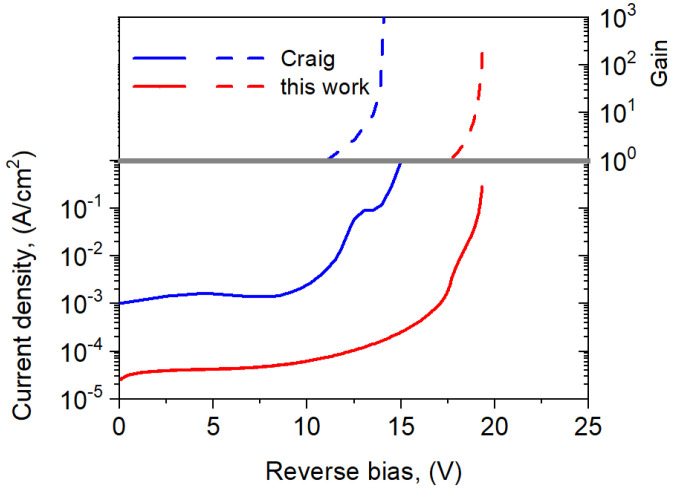
Comparison of the simulated theoretical *I*-*V* characteristics and gain for the analyzed npBp APD at *T* = 300 K with data according to [15].

Figure 11 shows a comparison of the theoretically simulated *I*-*V* characteristics for the analyzed npBp barrier detectors from our work with literature data according to Ref. [15]. The presented literature data do not correspond to the analyzed structure, i.e., they differ in the stoichiometric composition of the AL and its doping level as well as in the ML material and geometrical dimensions. The In_0.22_Ga_0.78_As_0.19_Sb_0.81_ absorber had a thickness of 1.4 µm and a *p*-type doping level of 1 × 10^16^ cm^−3^. The ML was made from AlGaAsSb. Hence, this detector was characterized by a breakdown voltage of 15 V and a punch-through voltage of 12.5 V at a temperature of 300 K. In the work [15], IR illumination was carried out using an Eblana Photonics 2.1 μm fiber-coupled laser, but we calculated an illumination power of *p* = 10 W and a wavelength of *λ* = 1.55 μm. The gain was defined as the photocurrent ratio at a given voltage to the photocurrent at the punch-through voltage. Minor differences in illumination do not significantly affect the current gain. Figure 11 presents the npBp avalanche detector reaching a significantly (about two orders of magnitude) lower dark current and comparable gain to the detector presented in the literature.

## 4. Conclusions

This paper presents an analysis of the avalanche parameters of npBp IR barrier detectors based on a quaternary A^III^B^V^ InGaAsSb material, which was lattice-matched to the GaSb substrate and operated at 300 K. Despite small discontinuity in the conduction band, a significant impact of the charge layer on the detector avalanche parameters was demonstrated. The doping level of the charge layer influences the dark current and photocurrent and changes the multiplication gain and breakdown voltage. As the doping of the charge layer increases, the dark current and photocurrent increase but the excess noise factor and breakdown voltage decrease. The proposed detector structure allows for obtaining comparable gain and lower dark currents in relation to the APD detectors made of InGaAsSb previously presented in the literature. The performed simulations confirmed the possibility of developing avalanche detectors with high gain at room temperature made of quaternary InGaAsSb materials.

## Figures and Tables

**Table 1:** InGaAsSb material parameters used in simulation at *T* = 300 K.

**Parameters**	**Electron**	**Hole**
SRH lifetime (ns)	250	250
Radiative coefficient (cm^3^/s)	1.0 × 10^−10^	1.0 × 10^−10^
Auger coefficient (cm^6^/s)	1.0 × 10^−28^	1.0 × 10^−28^
Impact coefficient a (cm^−1^)	4.0 × 10^6^	2.2 × 10^6^
Impact coefficient b (V/cm)	1.8 × 10^6^	3.0 × 10^6^
Effective masses, (m_e_;m_h_)/m_0_;	0.036	0.415/0.035

**Table 2:** InGaAsSb-based barrier APD structure.

Layer	Material	Thickness (nm)	Doping Concentration (cm^−3^)/Type	Energy Gap (eV)	Name of Layer
8	GaSb	100	2 × 10^18^/p	0.72	Contact
7	Al_0.20_Ga_0.80_Sb	100	2 × 10^16^/p	0.93	Barrier
6	In_0.14_Ga_0.86_As_0.10_Sb_0.90_	2000	1 × 10^15^/p	0.56	Absorber
5	In_0.10_Ga_0.90_As_0.10_Sb_0.90_	100	1 × 10^15^/p	0.58	Gradient
4	In_0.10_Ga_0.90_As_0.10_Sb_0.90_	60	8 × 10^17^/p	0.58	Charge
3	In_0.05_Ga_0.95_As_0.10_Sb_0.90_	200	1 × 10^16^/p	0.62	Multiplication
2	In_0.05_Ga_0.95_As_0.10_Sb_0.90_	400	2 × 10^18^/n	0.62	Contact
1	GaSb	500	2 × 10^18^/n	0.72	Buffer

## Data Availability

The original contributions presented in this study are included in the article. Further inquiries can be directed to the corresponding author.
